# CRISPR/Cas9-based systems: taking recombineering to the next level

**Published:** 2016

**Authors:** Sina Adrangi


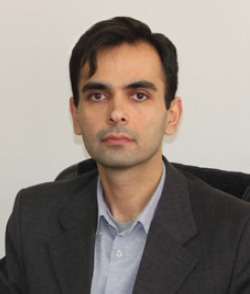


*Escherichia coli* is one of the most widely used hosts for recombinant protein production. In most cases, especially when high levels of target proteins are required, plasmid-borne expression is the method of choice for this purpose. The availability of a plethora of *E. coli* plasmid expression vectors with various promoters, selection markers, purification tags, fusion partners and auxiliary proteins has greatly simplified the process of cloning and expression in this microorganism. However, there are certain situations where integrating the gene of interest into the chromosome of the host may be advantageous over plasmid-based systems. For example, when the function of the target gene needs to be evaluated under physiological conditions, variations in plasmid copy number and, consequently, expression levels can affect the results and may complicate the interpretation of the obtained data (1, 2). Similarly, in metabolic engineering where expression stability is considered of primary importance chromosomal integration offers certain advantages (3). Even in cases when *E. coli* is being used simply as a host to obtain high levels of heterologous recombinant proteins for in vitro analyses, chromosomal integration of the corresponding gene under the control of a strong exogenous promoter may be used as a reliable alternative to plasmid-borne protein expression (4).

Several methods for the integration of the gene of interest in the genome of *E. coli* are available and both the site-specific and homologous recombination pathways have been exploited for this purpose. Methods based on site-specific recombination include the FLP/*FRT*, Cre/*loxP* and Int/*att* systems (2, 5, 6). In most cases, the enzymatic activity required for these processes are provided on helper plasmids. The gene of interest is inserted into an integrative plasmid containing the corresponding recognition sequence and a selectable marker and transformed into a host carrying the required enzymatic function. These systems can be used to integrate very large constructs. However, insertion can only occur in a limited number of pre-existing recognition sequences in the host genome (7). Methods based on homologous recombination, on the other hand, can be used to integrate the target gene into virtually any position in the host genome but are limited to smaller constructs. As the size of the construct increases, the efficiency of the integration decreases to the point that at construct sizes larger than 2.5-3.5 kb practically no recombinant clones can be obtained (7). Homologous recombination-based methods may employ the *E. coli* recombination machinery (8) or may depend on phage recombination systems such as the phage lambda Red system (9). The term recombineering is usually used to refer to chromosomal integration methods that utilize phage-derived recombination functions. Not surprisingly, in an attempt to overcome the aforementioned limitations, hybrid methods that use both the site-specific and homologous recombination systems have been developed by different research teams around the world. For example, recombinase-assisted genome engineering (RAGE) has been used to integrate a 34 kb construct into a predetermined location in the *E. coli* genome (2). The sophisticated and multi-step nature of these methods, however, has limited their practical applications. Thus, despite all these developments, the integration of a large construct into a desired location in the genome of *E. coli* still remains a daunting task. But this may be about to change.

The clustered regularly interspaced short palindromic repeats (CRISPR) system is part of a defense system in bacteria and archaea that together with CRISPR-associated (Cas) proteins detect and degrades foreign nucleic acids (10). This system integrates short sequences of foreign DNA into the host’s CRISPR locus which is then transcribed into small CRISPR RNAs (crRNAs) that after hybridizing with a trans-activating crRNA (tracrRNA) direct the degradation of invading DNA by Cas endonucleases. In 2013, a joint research team from the Rockefeller University and the MIT coupled the CRISPR/Cas9 system of *Streptococcus pyogenes* with the lambda Red recombineering system to introduce point mutations in *E. coli* and *Streptococcus pneumoniae* with an efficiency of 65-100% (11). Other research teams have expanded this technique to introduce gene-size insertion and deletions (12). The importance of such a high efficiency is twofold. First, it eliminates the need for selectable markers as transformants can easily be detected by colony PCR screening. Second, it implies that it may be possible to integrate constructs much larger than those possible with lambda Red system alone. In fact, a recent study has demonstrated that this technique is capable of integrating 10 kb constructs with an efficiency of approximately 50% (13). These methods are also far less complicated than other techniques and the whole process can be completed in a single day.

Although CRISPR-based recombineering techniques are still in the early stages of development, they have already produced astonishing results. These methods may finally provide biologists with the much needed tools for large scale manipulation of *E. coli* genome and, probably, revolutionize the fields of molecular genetics, metabolic engineering and synthetic biology.
